# Chitosan: Sources, Processing and Modification Techniques

**DOI:** 10.3390/gels8070393

**Published:** 2022-06-21

**Authors:** Alessandro Pellis, Georg M. Guebitz, Gibson Stephen Nyanhongo

**Affiliations:** 1 Department of Chemistry and Industrial Chemistry, University of Genova, Via Dodecaneso 31, 16146 Genova, Italy; alessandro.pellis@unige.it; 2 Department of Agrobiotechnology, IFA-Tulln, Institute of Environmental Biotechnology, University of Natural Ressources and Life Sciences, 1180 Vienna, Austria; guebitz@boku.ac.at; 3 Department of Biotechnology and Food Technology, Faculty of Science, University of Johannesburg, Johannesburg P.O. Box 17011, South Africa

**Keywords:** chitin, chitosan, deacetylation, chitosan modification, chitosan processing

## Abstract

Chitosan, a copolymer of glucosamine and *N*-acetyl glucosamine, is derived from chitin. Chitin is found in cell walls of crustaceans, fungi, insects and in some algae, microorganisms, and some invertebrate animals. Chitosan is emerging as a very important raw material for the synthesis of a wide range of products used for food, medical, pharmaceutical, health care, agriculture, industry, and environmental pollution protection. This review, in line with the focus of this special issue, provides the reader with (1) an overview on different sources of chitin, (2) advances in techniques used to extract chitin and converting it into chitosan, (3) the importance of the inherent characteristics of the chitosan from different sources that makes them suitable for specific applications and, finally, (4) briefly summarizes ways of tailoring chitosan for specific applications. The review also presents the influence of the degree of acetylation (DA) and degree of deacetylation (DDA), molecular weight (M_w_) on the physicochemical and biological properties of chitosan, acid-base behavior, biodegradability, solubility, reactivity, among many other properties that determine processability and suitability for specific applications. This is intended to help guide researchers select the right chitosan raw material for their specific applications.

## 1. Introduction

Chitosan is a copolymer composed of glucosamine and *N*-acetyl glucosamine derived from chitin. As summarized in many elegant reviews compiled in Table 1, chitosan is emerging as a versatile raw material for the synthesis and manufacturing of a wide range of products with application ranging from food, medical, pharmaceutical, health care, agriculture, industry, and environmental pollution protection. This is due to the reactive amino and hydroxyl groups which confer chitosan with many functional properties including polyelectrolyte, antimicrobial, antioxidant, gel-forming, biocompatibility, metal chelating and easy processability [1]. This impressive list of characteristics of chitosan is continuously and rapidly expanding its applications in many areas never imagined before. Chitin, the parent compound of chitosan, is a biopolymer present in many organisms including exoskeletons of the crustaceans (for example, lobster, shrimps, krill, barnacles, crayfish etc.), mollusks (for example, octopus, cuttlefish, clams, oysters, squids, snails), algae (for example, diatoms, brown algae, green algae), insects (housefly, silkworms, ants, cockroaches, spiders, beetles, brachiopods, scorpions) and cell wall of fungi (*Ascomycetes*, *Basidiomycetes*, and *Phycomycetes* for example, *Aspergillus niger*, *Mucor rouxii*, *Penicillium notatum*, *Trichoderma reesi* cell walls) [2,3]. Generally, the amount of chitin in these organisms ranges from 15–30% in crab cuticles, 20–30% crustacean exoskeletons [4], 30–40% shrimp cuticles, 5–25% insect cuticles [5] and 2–44% fungi cell walls [6,7]. Currently, the chitosan used for industrial application is mainly derived from crustaceans, especially crab, prawns and shrimp shells, whose exoskeletons are readily available as waste derived from the food processing industry. However, it is increasingly becoming available as a side-stream product from the breeding of cocoons from silk industry, a by-product of proteins extraction from insects for food/animal feed industries and fungal fermentation. Although fish scales are made of chitin, it is often discarded because its yield is very low constituting only 1 wt% of its whole weight [8]. According to some estimates, 10^12^–10^14^ tons of chitosan from crustaceans are produced every year [9] and the global market for chitin and its derivates was valued at US$2900 million in 2017, growing at a Compounded Annual Growth Rate (CAGR) of 14.8%) [2]. It is expected to reach US$63 billion by 2024 [2]. A reasonable number of companies including Chinova Bioworks, Heppe Medical Chitosan GmbH, Golden-Shell Biochemical and G.T.C. Bio-corporation are leading players in the market producing a wide range of products for food, drug, medical, textile and waste treatment with chitosan sourced from shrimp shell that occupies almost 80% of the total market [10].

One of the most prominent and well-established biomedical application of chitosan is producing homeostatic agents for wound management and wound healing promoting commercial products already approved by the United States Food and Drug Administration (USFDA) [10]. A number of companies are also emerging specializing in the production of fungi-based chitosan products including the Belgium-based company, KitoZyme whose products have already been recognized as safe by the Food and Drug Administration (FDA) and, the European Food Safety Authority (EFSA) for use in medical, food and beverage for example, in winemaking process (clarification, fining etc.) and dietary fiber, microbeads hydrogel for treating osteoarthritis [10]. Similarly, in Canada, Mycodev is producing chitosan for biomedical and pharmaceutical applications through fermentation while in China, Chibio is producing chitosan for food and pharmaceutical applications.

Given chitosan’s rapidly increasing industrial importance and ongoing intensive research, this review summarizes ongoing research in exploiting different sources of chitosan, extraction techniques and highlights some of the exploited functional properties based on source, extraction and processing techniques. This is intended to help guide researchers choose the right chitosan raw material for desired applications.

## 2. Biosynthesis of Chitin

**Figure 1:**
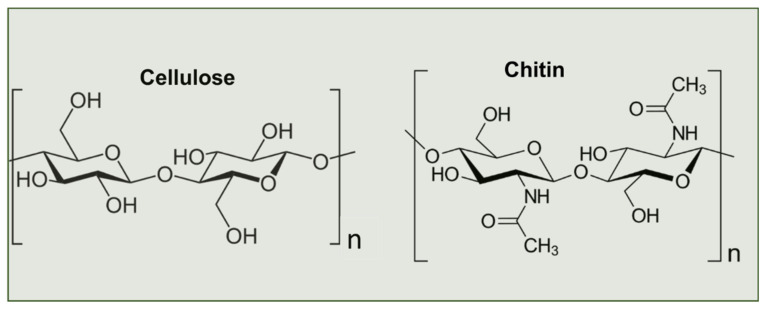
Structures of cellulose and chitin.

To understand the basis of exploitation of chitosan, it is important to briefly discuss various origins, chemical composition, similarities, and differences from different sources which influence not only the choice of extraction techniques but also the final properties of the chitosan. In Chitin, although similar to cellulose, the C_2_ hydroxyl (OH) groups of the glucose units are substituted with acetyl amine groups, hence termed poly-β-[1,4]-*N*-acetyl-d-glucosamine (Figure 1).

**Figure 2:**
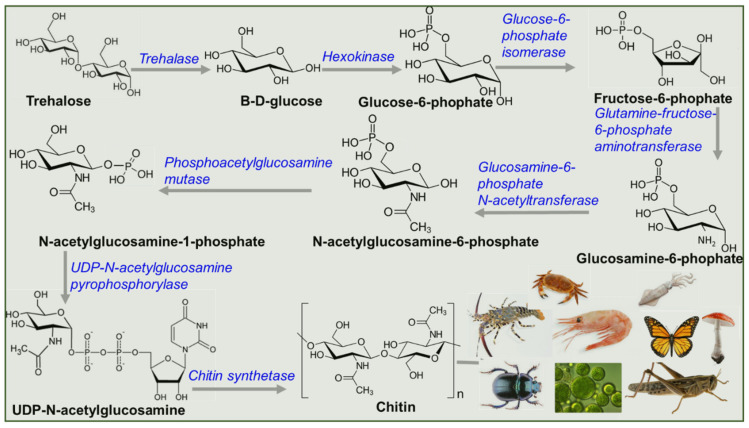
Chitin biosynthesis pathway.

The chitin biosynthesis pathway is highly conserved in all organisms, from algae to crustaceans and from fungi to insects. As summarized in Figure 2, chitin biosynthesis pathway can be generally divided into five distinct phases namely; (1) synthesis of *N*-acetylglucosamine-6-phosphate from sugars such as glucose, glycogen or trehalose via the hexosamine pathway [75], (2) synthesis of amino sugar uridine diphosphate *N*-acetylglucosamine-(UDP-*N*-acetylglucosamine), (3) polymerization UDP-*N*-acetylglucosamine by the action of chitin synthase into chitin and (4) deposition of chitin along the cell membrane and release into the extra-cellular space and finally, (5) assembly into chitin nanofibrils [76,77,78]. As elegantly summarized in previous reports [76,77,78] and shown in Figure 2, trehalose is first converted into glucose by trehalase, and the glucose is further converted into glucose-1-phosphate by phosphorylase. The formed glucose-1-phosphate is then converted into glucose-6-phosphate by phosphomutase, which is further converted into glucose-6-phosphate by a hexokinase. The fructose-6-phosphate is then converted into glucosamine-6-phosphate by an aminotransferase using L-glutamine. The glucosamine-6-phosphate is converted into *N*-acetylglucosamine-6-phosphate by *N*-acetyltransferase using acetyl co-A as a substrate. Further phosphate group is moved from position 6- to 1-phosphate position by phosphoacetylglucosamine mutase. A pyrophosphorylase using triphosphate as the co-substrate converts *N*-acetylglucosamine-1-phosphate into UDP-*N*-acetylglucosamine. In the last step, chitin synthase uses UDP-*N*-acetylglucosamine to produce chitin.

**Figure 3:**
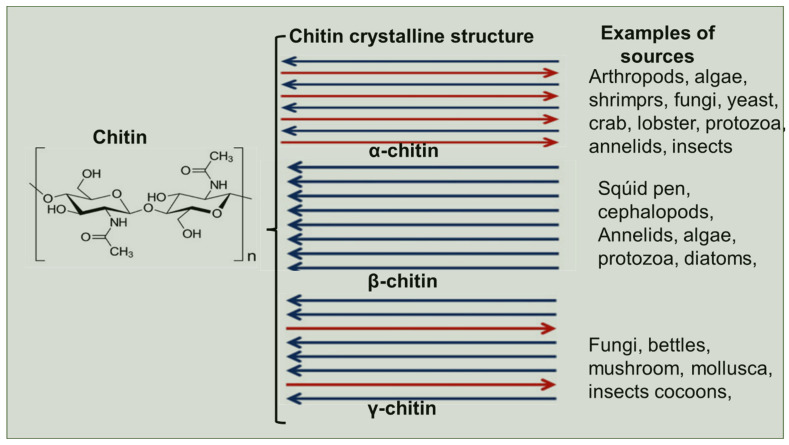
Orientation and arrangement of chitin microfibrils in *α*-, *β*- and *γ*-chitin [81].

The synthesized linear chitin chains are then assembled into microfibrils and organized in the extracellular matrix (cell walls, cuticles, peritrophic matrices) [79]. The composition of chitin varies between organisms, season, gender, age, habitat and other environmental conditions [80]. Based on X-ray diffraction studies, chitin microfibrils seem to orient themselves in three crystalline allomorphic forms namely; α-, β- and γ-chitin (Figure 3). These microfibrils also differ in orientation, number of chains, degree of hydration and unit size. The α-chitin crystalline structure is the most abundant form present in arthropod exoskeletons such as krill, lobster, crab, as well as in the insect cuticle [77].

The *α*-chitin is the most stable crystalline form of chitin. Each microfibril consist of approximately twenty single chitin chains arranged in an antiparallel to each other and resulting in a densely packed polymer with increased intra- and intermolecular hydrogen bonds that confers it a remarkable thermodynamical stability [2,78,82,83]. Unlike α-chitin, found mainly in arthropod exoskeletons, β-chitin is found in diatoms and squid pens microfibrils are organized in parallel [84]. This confers them with flexibility. The *γ*-chitin microfibrils found in fungi, yeasts, and insect cocoons [84], containing a mixture of parallel and antiparallel chains which confers them with both properties of *α*-form and *β*-form microfibrils [1]. The γ-chitin microfibrils show random chains as shown in Figure 3 [2,85]. Generally, chitin is also closely associated with other biological components, such as proteins, minerals, carbohydrates, lipids and pigments present in the shells. For example, fungal chitin contains α-chitin found in cell walls associated with glucans, and in insects they are embedded in a proteinaceous matrix.

## 3. Chitin Extraction Techniques

Chitin is found in association with other biopolymers in different organisms. For example, in fungi, chitin is covalently bonded directly or indirectly via peptide bridges to glucans in cell walls while in insects and other invertebrates, it is either covalently or none covalently associated with certain proteins. This variation implies that different extraction techniques maybe necessary. For example, insect and crustacean chitin forms part of the exoskeleton while in fungi chitin makes a complex flexible compound in cell walls covalently linked to glucans [86]. Further, chitin of marine organisms such as crustaceans are found associated with minerals mainly inorganic carbonate salts, chitin–protein complexes and also contains carotenoids (mainly astaxanthin) and lipids [87]. Although both arthropod and insect chitin are associated with proteins, lipids, minerals, pigments, their abundances vary. Generally, the shells of crustaceans contain 20–30% of chitin [88], 30–40% of proteins, 0–14% of lipids [89], 30–50% of minerals [90]. These percentages vary depending on the source, or even the species, from which chitin is isolated [91]. Distinct from crustaceans, insects generally contain 30–60% protein, 10–25% lipid, 5–25% chitin, 5–15% pigments and 2–10% minerals [84,92]. Fungal cell wall are flexible complex structures composed mainly of 2–44% [6] chitin chemically linked through α- and β-linkages to glucans (80–90%), 3–20% glycoproteins [2] and minor proportions of lipids, pigments, and inorganic salts [10].

Processes for industrial production of chitin from crustacean shell waste are well established exploiting the abundance of shells of crab, shrimp, and prawn from food processing industries. Crustaceans contribute 69–70% of chitin production [93]. The traditional extraction process involves various steps, namely, demineralization, deproteination, bleaching/discoloration and finally deacetylation to form chitosan as summarized in Table 2. Dissimilar to fungi and insect, the presence of minerals in crustaceans makes demineralization a crucial step. Demineralization is achieved through acid treatment using sulfuric, hydrochloric, nitric, acetic, oxalic and formic acids [94]. Mohan et al. [95], demonstrated that the use of hydrochloric acid during the extraction of chitin from insects produced chitin with superior quality as compared to other acids [96]. Acid treatment breaks down calcium carbonate into calcium chloride and carbon dioxide. While hydrochloric acid is the most preferred reagent for the demineralization of both insect and crustacean shells, attempts are being made to replace with more environmentally friendly organic acids [10]. Fungal extraction does not require the demineralization step but requires deproteinization using bases and strong acids at high temperatures and further neutralization [97]. A typical extraction process of chitin from fungi requires first treatment with alkali, usually 1 M NaOH at 60–120 °C for 0.5–12 h), to remove proteins, lipids, and other alkali-soluble carbohydrates [98]. The remaining alkali insoluble material containing mainly chitin is further treated with acids such as 2–10% acetic acid at 50–95 °C [99] in order to remove acid soluble material. The obtained acid soluble material, rich in chitosan, is then treated with alkali up to 2 N NaOH followed by centrifugation and washing with acetone and ethanol [100,101], followed by centrifugation and washing with acetone and ethanol [98]. Alkaline conditions degrade cell wall material resulting in insoluble proteins and chitin which is then further treated with an acid such as hydrochloric, lactic or acetic acid. Acetic acid is preferred for effectively removing phosphates and insoluble materials. It should be noted that high alkali concentration can cause chitosan oxidation, extensive chain degradation especially at high temperatures and long deproteinization incubation time. Similarly, acid treatment can also affect the final yield of chitosan during the extraction process. Lactic acid produced higher yield of chitosan than hot sulfuric acid even under lower temperature [98], formic acid (6% *v*/*v*) gave a higher yield of chitin compared with acetic acid [99]. Although hydrochloric acid causes a greater extent of hydrolysis of the acetyl moieties, it produces chitosan with higher DDA compared with acetic and formic acid [9]. Generally increasing the concentration of acids results in increased DDA and darker colored chitosan [9]. This procedure does not include the deproteinization nor the demineralization processes required in the extraction of chitosan from crustacean sources. The fungal chitin extraction process has been shown to result in chitin free of proteins that could cause allergic reaction, making them suitable for biomedical applications [102]. Extraction of both marine and fungal sources may require discoloration or bleaching to remove pigments that are naturally present in the organism [6]. Discoloration is easily achieved using organic solvents for example, acetone while bleaching is achieved using sodium hypochlorite or hydrogen peroxide [103]. For example, decolorization of crustacean chitin is achieved using bleaching agents such sodium hypochlorite potassium permanganate and oxalic acid or hydrogen peroxide, while a mixture of methanol–chloroform or alcohol–chloroform has been found effective for decolorizing insect’s chitin [84].

Besides chemical-based chitin extraction techniques, several other methods including biobased methods, the use of ionic solvents, deep eutectic solvents and ultrasound-assisted techniques summarized in Table 2 are emerging suitable methods too. Microbial fermentation technology that employs lactic acid producing microorganisms or the use of biologically produced organic acids are proving efficient systems to obtain high quality chitin. Biological extraction processes either use fermentation processes exploiting the ability of such microorganisms like *Lactobacillus* for example, *L. paracasei*, *L. plantarum*, and *L. helveticus* to produce organic acids.

These acids are efficient in demineralizing chitin. Microbial fermentation using Aspergillus sp., Pseudomonas sp., and Bacillus sp. has also been shown to be effective [143]. Biological deproteinization process uses proteases produced by microorganisms. Approximately 95.3% deproteinization and 99.6% demineralization is achieved without comprising the quality of chitin [111]. Among the enzymes, microbial proteinases, fish entrails proteases for example, intestines of sardinella (Sardinella aurita) and grey triggerfish (Balistes capriscus) [144] have proved useful deproteinization agents [105]. Enzyme based methods share the same demineralization mechanism with chemical methods. Nevertheless, despite promising, biological extraction approaches suffer from commercial scalability. However, intensive research is ongoing to make this approach industrially feasible. Other emerging extraction techniques include the use of ionic liquids, deep eutectic solvents (DES), microwave, ultrasound, and pulsed electric field technologies for the deproteinization [142]. Of these approaches, microwave-assisted extraction, ionic liquids, deep eutectic solvents and ultrasound-assisted extraction [133,145] offers enhanced process control, energy-efficiency, and cost-effectiveness [130,141] in comparison with the conventional chemical approaches. Especially the use of emerging green solvents (ionic liquids and deep eutectic solvents (DESs)) have been applied to many fields, such as biomass for separation and purification, pretreatment and synthesis of polymers including chitin extraction. For example, the use of ionic liquid 1-ethyl-3-methylimidazolium acetate ([C_2_mim] [OAc]) resulted in successful extraction of chitin from shrimp, fly larva, crab and lobster with different properties, reconfirming specie dependence [146]. Ionic liquids (ILs) are salts generally composed of a large organic cation and a smaller organic or inorganic anion, possessing a melting temperature below 100 °C. Chitin dissolution is complex and depends not only on the strong hydrogen bond acceptor ability of the IL anion and its interaction with the cation, but also on the chitin type and degrees of acetylation and crystallinity [122]. Regarding chitin, the highest solubility was reported for [C_2_C_1_im] [CH_3_COO], being approximately 20 wt% with microwave irradiation [147]. The extraction of chitin from crustaceans using ILs focused mainly on its complete dissolution followed by the selective precipitation of chitin to obtain clean achieving a maximum of 94 wt% chitin yield from crustacean shells with [C2C1im] [CH3COO] [121]. Scaling-up the process was also successful, leading to the establishment of a company 525 Solutions at industrial scale [122]. Nevertheless, due to the toxicity and non-biodegradability of ionic liquids, deep eutectic solvents (DESs) are emerging as alternative with similar properties to those of ionic liquids. DESs are a mixture of an acid and a base formed through complexing a hydrogen bond acceptor (HBA), usually a quaternary ammonium salt, with a hydrogen bond donor (HBD) or a metal salt. They are emerging as a new class of ionic liquid analogues derived from inexpensive commercially available raw materials with a melting point lower than that of each individual component. DESs are biodegradable, cheap and easy to produce [123]. Compared with traditional ionic liquids, they are cheap, environmentally friendlier and easy to prepare [148]. DESs contain hydrogen bond donors (HBD) and hydrogen bond acceptors (HBA) that posses a strong hydrogen bond interaction and electrostatic interaction. Normally, DESs are two-component or three-component systems. They are mixtures of quaternary ammonium salts, metal salts (for example, choline chloride, betaine) while HBD such as polyols, polyacids, and polyamines (for example, ethylene glycol, lactic acid, oxalic acid, urea) [133]. The DESs are able to preform both demineralization and deproteinization. For example, demineralization of chitin by choline chloride–malic acid is attributed to malic acid leaving the proteins and chitin weakening of the linkages within the inner structural organization of the shrimp shells [123]. The demineralization is realized through releasing hydrogen ions from DESs which react with the calcium carbonate in crustaceans resulting in solubilization and formation of calcium salts, water and carbon dioxide [129]. The removal of the calcium carbonate from crustaceans results in a less tight chitin-protein polymer. The DESs strongly interact with protein hydroxyl, carboxylic and amine groups consequently resulting in interruption of intra- and intermolecular hydrogen bonds within the network of chitin-protein fibrils and subsequent separation of chitin-protein fibrils [129]. Although the DESs have been observed to dissolve up to 9 wt% of chitin for example, in ChCl/thiourea used in 1/2 molar ratio under 100 °C, the dissolved chitin could easily recovered by using water or ethanol [132]. The extraction with ionic liquids produced chitin from shrimp with the strongest fibers, while weaker fibers were obtained with crab and lobster chitin although the latter were twice as elastic and, fly larvae chitosan were the weakest and least elastic fibers [146]. Besides crustaceans and fungi, another potential emerging source of chitin is that from insects. Insects constitute over 900,000 species out of the total 1.3 million different species on the earth [5]. Increasing demand for insects as an excellent alternative source of protein will eventually lead to increased availability of their chitin. By 2016, more than 120 companies had been registered farming insects for animal and human nutrition. By 2019 more than 6000 tons insect black soldier fly and the yellow mealworm protein meal was produced in Europe alone [149]. Many species of insects including honey bees, silkworms, and synanthropic files can be artificially reared and used as a promising new chitin source for industrial purposes [150]. Insect’s chitin extraction procedures are similar to those applied for crustacean sources except that insect chitin contains very low quantities of mineral when compared to crustacean shells [151]. This simplifies processing of insect chitin for application in the biomedical and pharmaceutical industries. A comparative study of chitin in exoskeleton of seven orthopteran species (Aiolopus simulatrix, Aiolopus strepens, Duroniella fracta, Duroniella laticornis, Oedipoda miniata, Oedipoda caerulescens, Pyrgomorpha cognate) showed that chitin content varied between 5.3 to 8.9% [152]. In summary, it is important to note the harsher the chemical extraction techniques employed during demineralization, deproteination and discoloration treatments with regards to chemicals used, pH, temperature and incubation time, the higher the degree of hydrolysis and may affect the quality of obtained chitin. Despite the advances in developing new environmentally friendly, efficient chitin extraction techniques, chemical extraction techniques remain, to date, the preferred routes due to the availability of chemicals and the possibilities of scalability.

## 4. Chitin Deacetylation Techniques

Deacetylation converts chitin into chitosan. The process involves the removal of the acetyl groups attached to amino group to expose the -NH_2_ groups. The degree of acetylation (DA) of chitin is a significant parameter influencing the biological, physicochemical, and mechanical properties and an important parameter that determines its classification whether it is chitin or chitosan. The deacetylation process results in a polymer containing both *N*-acetyl-glucosamines and glucosamines units. If the deacetylation produces a polymer with >50% *N*-acetyl-glucosamine units, it is still referred to as chitin if it is lower, than it is termed chitosan. Thus, deacetylation does not only affect acid-base behavior, electrostatic characteristics, biodegradability, self-aggregation, solubility, sorption properties, ability to chelate metal ions among many other properties but also determine its classification and affect its suitability for specific applications [95]. The percentage of *N*-acetyl-glucosamine units is termed the degree of acetylation (DA) and can vary from 50% to 100%.

During the deacetylation process, random depolymerization also occurs due to the extreme process conditions (for example, strong base, high temperatures and pressures) leading to the production of chitosan with varying chain length and water-solubility properties. Although chitin can be deacetylated using either acids or alkalis, glycosidic bonds are very susceptible to acid hydrolysis therefore alkali-deacetylation using NaOH at high temperature is increasingly being used more frequently to avoid unwanted chain termination [96]. Satisfactory deacetylation is achieved with concentrated NaOH or KOH (40–50%) at temperatures above 100 °C [59,153]. This industrial approach hydrolyzes the amide bonds makes it possible to produce several chitosan products in the form of flakes, fine powder, beads, or fibers. Generally, the extent of deacetylation is the function of concentration of NaOH, reaction time, temperature, density, and molecular weight of the chitin initial polymer [59]. Approximately 82% deacetylation is achieved during treatment of chitin with 50 wt% NaOH for 1 h at 100 °C [59,95]. This process can lead to chitin with DDA as low as <10% and the molecular weight as high as 1–2.5 × 10^6^ Da corresponding to a degree of polymerization of ca. 5000–10,000 and chitosan with DDA ranging from 40% to 98% and the molecular weight ranges between 5 × 10^4^ Da and 2 × 10^6^ Da [59]. However, chitosan generally has a DDA between 13 and 40% and molecular weight (M_w_) between 2 × 10^5^ to 1 × 10^6^ Da [153]. It is important to note that due to the higher reactivity of β-chitin it is much easier to destroy its crystalline structure compared to α-chitin during deacetylation because of the loose arrangement of chitin molecules completely converting it into amorphous unlike highly crystalline chitosan from α-chitin [154]. Generally, alkaline deacetylation consumes high quantities of energy; large amounts of alkali solution produce chitosan with varied DDA and broad M_w_ distribution [155]. As noted by Jug and Zhao, several studies have found that the chemical treatments alter the structural properties of chitin, due to swelling, dissociation of hydrogen bonds, and rearrangements of polymeric chains, and the different forms of chitin responded differently such as weakening inter-sheet hydrogen bonds and decreasing crystallinity index [156]. Alkali- or acid treatment of β-chitin converts them into α-chitin, that affects its original functional properties, its high reactivity and susceptibility toward solvents [157]. Strategies to minimize chain degradation include avoiding the use of acids which easily hydrolyze glycosidic bonds, reducing the amount of alkali added by using water miscible solvents like 2-propanol or acetone [155], or reducing deacetylation reaction time.

Alternatively, other gentler extraction techniques such as microwave-assisted extraction, combined steam explosion and deep eutectic solvents integrated with microwaves and enzymatic deacetylation techniques are emerging as highly promising also environmentally friendly processes to produce chitosan. Microwave-assisted chitin deacetylation using sodium hydroxide increased deacetylation efficiency beyond >90% in 3 h as compared 21 h during conventional alkali treatment. Steam explosion has also been shown to facilitate deacetylation of chitosan [158]. During steam explosion, chitin is treated in a puffing gun with saturating steam at increased pressure and temperature for several minutes followed by explosive decomposition. The conversion of the steam energy into thermomechanical force breaks the intermolecular interactions of molecules and frees chitin. Chitin with 75% moisture content exhibited maximum DDA (43.7%) when compared to chitin containing 50% and 35% of moisture which resulted in only 40% and 32% DDA [158]. Chitin extracted by deep eutectic solvents had high purity (74–91.345) and yield (12.71–26%) compared to the conventional acid/alkali method (purity 91% and yield 6.5%) [158]. Further combining microwave with deep eutectic solvents resulted in effective deproteinization efficiency (88–93% rate of removal) in shrimp chitin [159]. Further, enzymatic deacetylation using chitin deacetylases obtained from different biological sources such as fungi and insects [90,160] present an efficient alternative strategy [160].

Various proteinases and deacetylases are emerging as competent technologies for deproteinating and deacetylating chitin [105]. Chitosan deacetylases are mainly derived from bacteria, fungi and a few insects. Among the prominent fungal chitin deacetylases are those produced by Mucor rouxii, Absidia coerulea, Aspergillus nidulans and Colletotrichum lindemuthianum [161]. It is important to note that the different deacetylases show different catalytic efficiency. Chitinolytic hydrolyzing enzymes are classified according to their mode of action into endo- and exochitinases can completely hydrolyze chitin. The endo-chitinases hydrolyze internal glycosidic bonds producing fragment ranging from dimers to polymers while the exo-chitinases act on the non-reducing end of chitin releasing monomeric and dimeric *N*-acetyl glucosamine units. For example, a deacetylase from *M. rouxii* performs sequential exo-type deacetylation at the non-reducing end of the oligomer, while deacetylase from C. lindemuthianum hydrolyzes a single acetyl group before dissociating and forming a new active complex [162]. Generally, most of the bacterial chitosan deacetylases preferably act on low-molecular-weight chitosan. Of all the chitin deacetylases, those obtained from from *Rhizobium* spp. and Vibrio cholerae [162] are known to efficiently produce chitosans [163,164]. Due to chitosan´s similarity with carbohydrates such cellulose, it is also important to note that enzymes such as cellulases and lysozymes are also able to hydrolyze chitosan [162]. Indeed, chitosan oligomers with 5–30 kDa have been produced using cellulases, pepsin and lysozyme [165]. The enzymatic deacetylation process tends to produce homogenous chitosan, although this approach is currently not industrially feasible due to the use of high cost of enzymes [90]. However, it should be noted that chitin deacetylases are not efficient in deacetylating insoluble chitin. It is therefore important to pretreat chitin.

## 5. Structure-Function Properties of Chitosan

### 5.1. Influence of DDA and Molecular Weight (M_w_) on Chitosan Properties and Applications

The DDA, polydispersity and M_w_ of chitosan are the most significant parameters influencing its biological, physicochemical, mechanical properties and hence its application. For example, DDA and M_w_ influences solubility, reactivity, acid-base behavior, electrostatic behavior, flexibility, polymer conformation, viscosity, crystallinity, porosity, tensile strength, conductivity, ability to chelate metals and photoluminescence. Moreover, the same two parameters listed above (DDA and M_w_) also affect many biological properties such as biodegradability, biocompatibility, mucoadhesion, hemostatic, analgesic, adsorption enhancer, antimicrobial, anticholesterolemic, antioxidant among many other properties that determine the material’s suitability for specific applications [10]. When protonated, the -NH_2_ group enables chitosan to make complexes with negatively charged derivatives for example, proteins, dyes, enzymes, tumor cells, bacteria cell wall proteins, DNA, RNA as well as various metal ions its neutral or negatively charged hydroxyl groups of D-glucosamine [10]. Under certain conditions, its insolubility at neutral and solubility under alkali conditions makes it a versatile polymer for application in polymer synthesis, solution or as solid polymer [10]. Generally, a high number of acetyl groups prevents chitosan’s enzymatic degradation (by enzymes such as lysozyme), making it suitable for producing drug delivery systems [166]. Although chitin/chitosan is readily soluble in many organic solvents and dilute organic acid solutions such as acetic acid and formic acid, its poor solubility in water has been one of the hindering challenges towards its full exploitation. To this effect many studies have been developing techniques to enhance the solubility of chitosan in water [9]. Acid hydrolysis with concentrated hydrochloric acid conducted at 80 °C produces chitosan oligomers with a degree of polymerization between 1 and 40 [9]. Nitrous acid treatment is also effective resulting in selective, rapid, and easily controlled, stoichiometry products. Nitrosating agents instead attack the glucosamine but not the *N*-acetylglucosamine moieties and cleave the glycosidic linkage. Chemicals such as hydrogen peroxide and hot phosphoric acid are also used. The use of acids produced in the human body such as acetic acid, HCl, lactic acid, citric acid, and pyruvic acid can potentially be also used to solubilize chitosan in water 9 except phosphoric acid) [167]. A Second strategy involves the deacetylation combined with hydrolysis of long chain chitosan polymer into lower M_w_ oligomers. Increasing the amino groups during deacetylation and whilst, respectively, decreasing the acetyl groups lead to enhanced solubility. This is because, under acidic pHs below 6, the amino groups are fully protonated. However, increasing pH beyond 6 gradually decreases its solubility due to deprotonation of the amino groups [168]. Increasing the DDA leads to oligomers with higher protonated amino groups which facilitate its solubility.

Thirdly, chitosan M_w_’s affect its water-solubility properties due to the presence of free amine without need of acidification [169]. Acid hydrolysis also leads to chitosan with decreasing M_w_ while concomitantly increasing its solubility [105]. Chitosan with M_w_ < 30 kDa is easily water-soluble without adding any acid. Solubility of chitosan with M_w_ between 22 and 30 kDa can be enhanced by adding acid [105]. This also applies to chitosan with M_w_ > 30 kDa. When the M_w_ of chitosan is above 30 kDa, protonation of its amino groups becomes a prerequisite to dissolve it in water. It is known that chitosan M_w_ lower than 2 × 10^6^ Da and containing 7% w/w nitrogen is suitable for textile, food, photography, medical and environmental applications [170]. However, although deacetylation is crucial in converting chitosan into water soluble oligomers, higher M_w_ tend to increase inter- and intra-molecular hydrogen bonds between chitosan chains leading to poor solubility [171]. High Mw chitosan ranges from 310 to 375 kDa [172], medium Mw ranges from 190 to 310 kDa (O’Callaghan & Kerry, 2016), and low Mw is below 90 kDa [173]. For example low MW chitosan is mainly preferred for drug delivery applications. Chitosan hydrolysis can easily be achieved using several hydrolytic enzymes including lysozyme, chitinases, some cellulases and lipases [59] and chemically using HCl, HNO_2_, H_2_O_2_ and potassium persulfate while sonication, electromagnetic irradiation, gamma irradiation, microwave irradiation and thermal treatment constitute the commonly used physical processes. The DDA and solubility properties of chitosan influence their functional properties and application. For example, chitosan with higher DDA is suitable for making films with higher tensile strength, water transmission while membranes with DDA between 65 and 80 were effective in inducing inflammation reactions [174]. Chitosan with a DDA of 45–55% is highly soluble in water and weak acid [175] making it suitable for making flexible and transparent materials [176]. As summarized in altering the DDA changes the biological functions of chitosan into antibacterial anti-tumor, anti-inflammatory, wound healing properties, immune activation [106]. Similarly, chitosan DDA also found to influence biocompatibility, biodegradability, hydrophilicity, muco-adhesion, hemostatic, analgesic, anticholesterolemic, antioxidant, and adsorption-enhancing properties of chitosan-based biomaterials [174]. For example, chitosan sponges produced from chitosan with different DDA and Mw shows that cell spreading was much higher on sponges made with higher DDA which led to increased activities of alkaline phosphatase, osteopontin, vascular endothelial growth factor-A (VEGF), interleukin-6 (IL-6), and reduction in monocyte chemoattractant protein-1 (MCP-1), sclerostin (SOST) and dickkopf related protein-1 [177]. Sponges made from chitosan with lower DDA increased secretion of osteoprotegerin and SOST as compared to higher DDA while a combination of high DDA and Mw increased secretion of VEGF and IL-6, reduced the secretion of osteopontin as compared to chitosan with similar DDA but with lower Mw [177]. These observed variations clearly indicated the possibility to introduce desired tailored conditions in tisuue engineering or wound management. Materials produced with DDA value > 70% have been shown to be suitable for making material suitable for drug delivery applications [178].

The DDA and Mw of chitosan has also been shown to influences its antibacterial properties. This is because the presence of positively charged amino group that interact with the negatively charged bacteria membrane depends on concentration of -NH_2_ reactive free groups. Low molecular chitosan inhibited *Escherichia coli* and *Pseudomonas aeruginosa* [179] and *phytopathogens* [179,180,181]. The -NH_2_ groups alter the bacterial surface morphology which result in increased membrane permeability and loss of intracellular substances [182]. Commercially available chitosan antimicrobial compounds include HidroKi^®^, Axiostat^®^, Chitopack^®^, Tegasorb^®^, and KytoCel^®^ CWD [11]. Chitosan having a lower M_w_ of approximately 2 × 10^6^ Da and 7% *w/w* nitrogen is suitable for textile, food, photography, medical and environmental applications while because of their stiffness and higher mechanical properties, chitosan with high crystallinity higher are good for making tissue engineering platforms [183]. Higher DA makes chitosan less senstive to enzymatic biodegradation making them useful as delivery systems [166]. In contrast, low M_w_ chitosan is also suitable for producing efficient protein-based delivery systems for transport and release of intestinal drugs and bioactive compounds. Low M_w_ chitosan (<300 kDa) is suitable for the synthesis of wound dressings, food preservation materials, wastewater treatment, molecular imprinting and chelating materials. Chitosans with DDA (70–80%) and high M_w_ (>300 kDa) is recommended for the synthesis of drug delivery systems, scaffold materials for tissue engineering, cell and enzyme immobilization platforms, encapsulation, food packaging while chitosan with DDA (70–90%). Chitosan with low DDA (55–70%) and high molecular weight (>300 kDa) is suitable as emulsifying agent and for application in various pharmaceutical applications, synthesis of nanoparticles and application in food formulations. In contrast, chitosan with low DDA (55–70%) and low M_w_ (<300 kDa) is suitable for gene and drug delivery, plant protection and plant growth stimulator [2]. Chitosan with moderate M_w_ has higher anti-cholesterol activity [184]. Generally it has been observed that increasing DDA enhances stronger biological effects, decreasing the M_w_ generally increases the bioactivities [96], especially when the M_w_ is lower (for example, <20 kDa) than higher (for example, >120 kDa) [185]. The DDA, DA and M_w_ are therefore very important characteristics to consider when using chitosan for specific applications.

### 5.2. Influence of Origin of Chitosan 

In addition to DA, DDA and M_w_, the source (origin) of chitosan also influences its application. For example, comparing the three allomorphic forms of chitosan namely α-, β-, and γ-chitosans, shows that β-chitosan has higher solubility than the α-chitosan. This is attributed to the weaker binding forces among the chains of the β-chitosan. Due to their higher crystallinity, α- chitosan is not only less soluble but also stiffer. This stiffness confers it with higher mechanical strength, which makes it suitable for producing tissue engineering platforms. The higher reactivity of β-chitosan derived from squid pen than α-chitosan due to its hydrated structure and weaker intermolecular hydrogen bonds makes it more suitable for the synthesis of thin films, medical, food applications and biosensors products [186]. The higher solubility of β-chitosan compared to that of α-chitosan is attributed to weaker binding force that enhance its biological activities. Furthermore, squid pen chitosan with 31–49% DDA is free from calcium carbonate, carotenoids, and minerals which makes it suitable for biomedical applications [186]. γ-chitin contains both α- and β forms and hence properties from both forms [1]. However, low availability remains a barrier for its mass production and commercial application. The occurrence of chitosan of high purity and concentration in fish makes it more attractive for biomedical and pharmaceutical applications than chitosan of microbial origin [187]. The α-chitosan derived from marine crustaceans was the first most abundant chitosan readily available in larger amounts from food processing industries. It was broadly used for the production of biomedical products, although there are increasing concerns due to the extra effort needed to ensure that they are free from heavy metals [85]. They are extensively used in medicine (drugs, wood management, artificial organs, membranes, anti-coagulant, anti-microbial agent, in tissue generation of artificial bones and skins) [11], pharmacology (fungicides and drug carriers) [188], food systems (preservatives, coatings, antimicrobial and antioxidant agents), and cosmetology (body creams, hair additives, and lotions) [189]. The higher M_w_ of crustaceans (approximately1.5 × 10^6^ Da) makes it poorly soluble at neutral pH values, resulting in highly viscous solutions than that of fungi (1–12 × 10^4^ Da) and makes it attractive as thickening agent and for making tissue engineering platform and film [190]. Contradictory as it might seem, the presence of protein, lipid and chitosan in marine crustaceans has also been seen as a source of nutrients that can be used in formulating functional foods for therapeutic applications [191]. Although crab chitosan (15–30%) is composed of high mineral (30–50%) and protein (15–50%), it exhibits excellent antioxidant properties, thereby generating interest for developing, products that combat oxidative damages caused by free radicals [192]. Another important crustacean, Krill, contains protein (72.9–75.8%), lipid (12–50%), and chitin (20–30%), is suitable for incorporation into food formulations and application in health [191]. Krill-based chitosan also have higher porosity which makes them suitable for sulfate modification [10]. The presence of protein, lipids, pigments, and CaCO_3_ associated with shrimp chitosan [190] is viewed as potential source of high value-complex added products. Shrimp has been demonstrated to possess potent antimicrobial activities against pathogenic microorganisms (*Staphylococcus aureus, Enterococcus faecalis, Enterobacter aerogenes*, and *E. coli*). Further, shrimps chitooligomers have been found effective as replacement for antibiotics in animal feed products [193]. Furthermore, chitooligomers (12.3%) from shrimps can be used in animal feed, replacing antibiotics, particularly when the focus is on developing antibiotic residue-free animal products [193]. Chitosan from fish is emerging a good source of high-quality chitosan for biomedical applications [187] and agrochemical industry [103]. However, the main problem has been its availability since it constitutes <1% of fish´s body weight.

However, increasing interest in insect as a new alternative source of protein is making its chitosan more attractive due to foreseen increasing availability. Chitosan obtained from the larvae of Chrysomya megacephala shows excellent antioxidant activity with an IC_50_ value of 1.2 mg/m [194] while chitosan from the larvae of Lucilia cuprina was shown to have superior anti-bacterial activity against Bacillus subtilis and Klebsiella pneumoniae [194]. The mealworm beetle chitosan was effective against Staphyloccocus aureus, Escherichia coli, Listeria monocytogenes and Bacillus cereus [195]. The ability of the insect chitosan to cause cell deformation and leakage of cell contents, which leads to the breaking of the cell. It has already been shown that honeybee chitosan is suitable as food additive for preserving food [196]. Beetles have also been shown to be a good source of chitosan (~36.6%) [196]. The superior elasticity of insect chitosan is advantageous in polymer production [197]. Chitosan derived from insects has been found suitable for agriculture application (seed coating, plant protection, gene transferring), and biomedical applications for example, drug delivery, and as biomedical platforms.

Another even more prominent and fast emerging source of chitosan are fungi. In fact, their abundance is ranked second, next only to insects. Fungal chitosan is increasingly becoming attractive due to easy production under controlled conditions through fermentation. Although generally insect-derived chitosan has similar properties to those obtained from crustaceans, the superior particle size uniformity, even distribution of acetyl groups. lower Mw and unique Mw homogeneity, viscosity distribution and the absence of heavy metals makes fungal chitosan suitable for wide applications [191,198]. Their unique uniformity of particle size and antimicrobial effect has found them preferred for application in water cleaning, beer-brewing, wound management and textile production [198]. Their viscosity which is 3–5 times lower and M_w_ (1–2 × 10^5^ Da) but higher DDA (70–90%) [190]), make them suitable for application in food, healthcare, and pharmaceutical industries [199]. For example, chitosan marketed by Sigma “Kitozyme” is isolated from Agaricus bisporus and is an ingredient for wound healing, biosurgery, cell therapy, drug delivery, and vaccines [200]. Chitosan-based edible food coatings have also been used extensively for extending the storability and quality of fresh and processed foods, owing to their antifungal and antibacterial activities [201]. Although yeast contain far less chitin, it has been found suitable for the development of stabilizers and emulsifiers for food and nutraceutical applications [202]. Yeast derived chitosan was successfully used as food stabilizer, emulsifier and for nutraceutical applications [202]. Mucor indicus and Rhizopus oryzae chitosans were demonstrated to be effective in controlling field infestation by the entomopathogenic fungus (M. anisopliae). Interestingly, shiitake mushroom (*L. edodes*) chitosan has been shown to have a complex immune stimulant property [203]. “Kitozyme” isolated from Agaricus bisporus is used for making wound healing promoting agents, in biosurgery, cell therapy, drug delivery, and vaccines [201]. Aspergillus niger chitosan with residual glucans content lower than 2%, viscosity in 1% acetic acid higher than 15 Cps and settled density < 0.7 g/cm^3^ is the only chitosan allowed in winemaking since 2009 [204], aimed at reducing protein as an alternative to the commonly used bentonite as well as an antimicrobial agent. In summary, the lower viscosity, M_w_ and higher DDA of fungal chitosan, makes it appropriate for application in food, beverage, healthcare, and pharmaceutical industries [199].

Although algal chitosan from marine sources for example, coralline algae Clathromorphum compactum matrix is less studied, the presence of collagen makes them attractive for cell immobilization applications and as food additives [205]. Further, the α-chitosan nanofibrils from microalgae (Phaeocytis globosa) showed tensile strength comparable to β-chitosan nanofibrils obtained from squid (Loligo bleekeri) and tubeworm (Lamellibrachia satsuma), making them good candidates for making tissue engineering scaffold materials [206].

## 6. Tailoring Chitosan for Specific Applications

**Figure 4:**
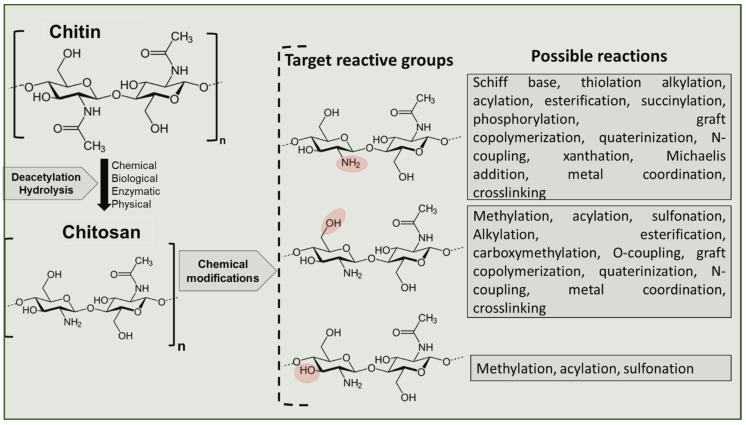
Target functional chitosan molecules (–OH, –NH_2_) groups for chemical modification.

The presence of reactive amino groups on C_2_ position, primary hydroxyl group on position C_3_ and the secondary hydroxyl group on position 6, offers a myriad of possibilities for modifying and exploiting chitosan for various applications through reactions summarized in Figure 4. These reactions have been extensively summarized in [204,207,208,209,210,211] to which the reader is referred to for detailed reactions. However, here we present in brief possible reactions targeting each functional group highlighted in Figure 4. For example, amino group targeted modification is achieved through such reactions as alkylation, acylation, quaternization, phosphorylation, acylation, nitration, sulfonation, xanthation, *N*-succinylation, thiolation, and graft copolymerization etc. while the hydroxyl groups are mainly modified through o-acetylation, sulfonation, methylation, hydroxylation, cross-linking/grafting [88]. Of these reactions, acylation, alkylation, carboxymethylation, *N*-phosphomethylation and Michael addition, quaternisation, carboxyalkylation, hydroxylation, phosphorylation, sulfation, and copolymerization are the mostly commonly used approaches. Amino group targeted substitutions reactions for example, with quaternary ammonium produces hydrophilic chitosan derivatives extensively studied for their antimicrobial, hemostatic. anticoagulant, hydrogel, film forming properties [212]. This approach is achieved firstly by turning the -NH_2_ into quaternary ammonium salt, introducing quaternary ammonium compounds or quaternary phosphonium compounds resulting in products with improved water solubility [213]. One of the most important quaternary chitosan derivative achieved through this process is *N*,*N*,*N*-trimethyl chitosan chloride with excellent solubility in aqueous solution which has found applications as a fluid absorption enhancer, antibacterial agent and gene vector, improving bioadhesion, biocompatibility, solubility, viscosity and swelling index properties of obtained polymers [213,214]. Amino group targeted phosphomethylation produces products with improved solubility, bactericidal, heavy metal chelating and tissue engineering properties) while *N*-targeted modification with *N*-methylene phosphonic, *N*,*N*-dicarboxymethyl, *N*-[(2-hydroxy-3trimethylammonium) propyl] produces soluble chitosan [211]. Selective amino group substitutions reactions while protecting –OH groups in the C_3_ and C_6_ of chitosan is an effective strategy for synthesize homogeneous *N*-quaternarised chitosan derivatives without *O*-methyl substitutions and *O-*silytation [211].

Dual functionalized chitosan hemostatic wound dressings were formulated using varying ratio of quaternized chitosan and phosphorylated chitosan along with tannic acid which acted as adjuvant hemostat and a crosslinker and poly-ε-lysine to impart the elastic and adhesive properties [215]. In contrast, *N*-acylation produces hydrophobic for example, by grafting fatty acids through amidation of –COOH groups of fatty acids with –NH_2_ of chitosan. This chemical process uses such chemicals as acyl halide or acid anhydride in pyridine, chloroform/pyridine, or methanol/water/acetic acid. This reaction also leads to the O-alkyl chitosan the C_2_ and C_6_–OH groups. To avoid this, protection with trityl groups is necessary [204]. Two alkynoyl-chitosan derivatives that could serve as a useful tool for linking other molecules through click chemistry, one containing alkyl spacers which are soluble in organic medium and another spacer soluble in water were synthesized by introducing alkyne functionalities on the amino group of chitosan without the use of protection groups [216].

Hydroxyl group (–OH) targeted reactions usually require initially protecting the -NH_2_ group. This is usually achieved using phthalic anhydride that allows for regioselective processes for example introducing sugar branches that improve water solubility of chitosan [217]. The phthaloyl group is then easily removed by introducing an electron-withdrawing group (for example, –NO_2_, –Cl) into the phthaloyl aromatic ring that deprotect the amino group [218]. Products with antifungal activity, flexible films have been produced through this strategy [219]. It should also be noted that while the C_2_–NH_2_ or C_6_–OH groups are easily accessible and modifiable, steric hindrance of the C_3_-OH group often makes it is tricky to modify, although it is easily chemically modified through methylation, acylation, sulfation) [220]. Sulfonation produces chitosan with many bioactive activities such as antioxidant and anticoagulant properties. For example, chlorosulfonic chitosan has improved antioxidant activity [221] while sulfonation of chitosan for metallic implants increased hydrophilicity of the implants while decreasing calcium deposits [211]. Chitosan thiolation adding different compounds such cysteine, thiolactic acid, thioglycolic acid, homocysteine, thiobutylamidine, glutathione, etc. is used to produce films, hydrogels, and nanoparticles for biomedical and food applications while phosphorylation performed using phosphorous pentaoxide in methane sulfonic acid as a solvent result in chitosans with high water solubility and metal chelating important for application in tissue engineering, drug delivery intermediates and in food industries [222]. Carboxyalkylation of chitosan produces water soluble and amphoteric chitosan excellent water soluble, nontoxic, biocompatible and biodegradable polymers suitable for biomedical applications as antimicrobial agents, in biosensor, wound healing, food industry and bio-imaging [222]. O-alkylating chitosan makes it soluble in chloroform, ethanol, water and acetic acid, etc. [88] while phosphorylation, quaternization and carboxymethylation of chitosan significantly improve the solubility of this polymer in different solvents at ambient conditions.

Graft copolymerization/cross-linking are also very important strategies used to tailor and broaden chitosan applications. To achieve this, various redox initiator molecules such as Fenton’s reagent, ceric ammonium nitrate, ammonium and potassium persulfate, potassium diperiodatocuprate and ferrous ammonium sulfate, enzymes, and microwave irradiation and γ-irradiation are widely used [96,222]. Free radical initiated grafting is one of the mostly commonly used approaches. For example chitosan-graft-poly (*N*-hydroxy ethyl acrylamide) using potassium persulphate initiator [223], polyacrylonitrile-g-chitosan (PA*N*-g-CS) in the presence of an initiator ceric ammonium nitrate [224], binary grafted chitosan with two monomers [acrylamide and (2-methacryloyloxyethyl) trimethyl ammonium chloride] via γ-radiation [225] graft copolymer of chitosan with poly [2-(acryloyloxy)ethyl trimethylammonium chloride] in the presence of potassium persulphate initiator [226], chitosan-g-polyaniline in the presence of APS [227]. Similarly, various crosslinking molecules such as simple phenolic compounds, glutaraldehyde, epichlorohydrin, ethylene glycol, diglycidyl ether and sodium tripolyphosphate etc. are used [11,88,227,228]. Glutaraldehyde mediated cross-linking of chitosan by forming a Schiff base (imine) is the most studied technique which leads to condensation reaction between the aldehyde function and a primary amine group from the chitosan chain in the presence of labile hydrogen. Cross-linking using glutaraldehyde produces polymers with high adsorption tendency for several metal ions as follows: Cd > Cu > Ni > Ag > Pb > Zn [222]. Benzoyl chitosan biopolymers that play a significant role during drug delivery, cosmetics, wound healing management derived from the synthesis of o-benzoyl chitosan derivatives of benzoic acid and *p*-methoxybenzoic acid were produced in trifluoroacetic acid anhydride/phosphoric acid mediated acylation [222]. Recently chitosan-based hydrogels with fast gelling, tunable elasticity and mechanical properties were obtained through Schiff-base crosslinking of dialdehyde debranched starch with chitosan amino groups [229]. On the other hand, epoxidation reactions are used to obtained chitosan hardened polymers [206,230].

Radiation-induced modification of chitosan is an emerging toxic free alternative process to the use of chemical crosslinking molecules. Several studies have demonstrated the possibility of introducing a variety of functional molecules into chitosan backbone through radiation including synthesis of chitosan-g-maleic acid copolymers [231], grafting of acrylic acid, acrylamide and acrylonitrile on to chitosan via microwave radiation [232]. Similarly to radiation, enzymes are also emerging as the safest strategy to modify or synthesize chitosan-based polymers. Many chitosan derivatives with unique properties such as increased water-solubility, thermal stability, pH-sensitive, adhesive have successfully been synthesized. TEMPO/laccase redox system has been used to selectively oxidize the chitosan C_6_ group in order to generate water soluble chitosan [233], synthesizing chitosan-based hydrogels using lignin and simple phenolics as crosslinker [169,230,234]. For example, phosphorylase was used to produce amylose-grafted chitosan copolymers by reacting chitosan and α-d-glucose 1-phosphate [235]. Summarizing, there is no shortage developing strategies for tailoring chitosan for many industrial applications.

## 7. Conclusions and Future Perspectives

Significant progress is being made in developing technologies for producing chitosan from other novel and attractive emerging sources such as insects and fungi, thanks to advances in insect farming (insect biotechnology) and fungal fermentation processes. These new sources not only providing a new source of chitosan but rather chitosan with superior properties that can easily and safely be used in food and medical, pharmaceutical applications and overcome the challenges often encountered with marine based chitosans obtained as by-product of food processing industry. The observation that chitosans with different DDA and Mw greatly influence their inherent properties and, hence, their function increases the scope of tailoring it for specific applications especially in tissue engineering and wound healing process. The fast-increasing demand for chitosan associated with increasing understanding its properties, extraction techniques and increasing numerous ways of chemically modifying and tailoring its properties is significantly expanding field of applications of chitosan. Although marine crustacean chitosan has predominantly been used in industry due to huge availability generated from the food industry, fungi and insect chitosan will in future become more mainstream raw materials due to increasing availability driven by advances in biotechnological processes for their mass production. For example, the recent increase in exploiting insects and the fast-expanding insect biotechnology field, as a new source of protein for both human and animal feed and increasing fermentation technology knowledge in fungal biomass and excellent properties, are once again propelling chitosan into an important strategic raw material for the future. The real utilization potential of chitosan for industrial applications is only just starting.

## Figures and Tables

**Table 1:** Summary of major applications of chitin and chitosan in the different fields.

Field of Application	Applications	References
Biomedical and Pharmaceutical applications	Antioxidant: free radical scavenger/quencher Antimicrobial agent: positively charged chitosan-NH_2_ groups interact with negatively charged microbial cell membrane creating pores Drug delivery: mucoadhesive properties increase drug permeation of intestinal, nasal, and buccal epithelial cells, Gene therapy: Delivering various genes and siRNA Chitosan based drugs. For example, lowering effect of cholesterol for obesity treatment Regenerative technology/tissue engineering: bone, neural, cornea, cardiac and skin regenerative technology. Provides a three-dimensional tissue growth matrix, activate macrophage activity and stimulate cell proliferation Wound management: homeostatic agent, participate in repair, replacement, activation of humor immunity, complement system, and CD4+ cells, enhances granulation as well as the organization of the repaired tissues. It slowly degrades into *N*-acetyl-β-d-glucosamine that stimulates fibroblast proliferation, regular collagen deposition in addition to stimulating hyaluronic acid synthesis at the wound site.	[11,12,13,14,15,16,17,18,19,20,21,22,23,24,25,26,27,28,29,30,31,32,33]
Health care products	Cosmetics formulations: Antimicrobial, antifungal, UV absorbing abilities exploited in various cosmetics formulations including in shampoos, rinses, colorants, hair lotions, spray, toothpaste formulations and tonics. Sunscreens, moisturizer foundation, eyeshadow, lipstick, cleansing materials, and bath agent, toothpaste, mouthwashes, and chewing gum as a dental filler.	[34,35,36,37]
Food Industry	Packaging, edible coatings, body filling, emulsifying agent, natural flavor extender, texture controlling, thickening and stabilizing agent, food preservation (antimicrobial agent), antioxidant agent. Flocculation/Clarification and deacilification of fruits and beverages	[38,39,40,41,42,43,44,45]
Agriculture	Antimicrobial activities against various plant pathogens. Fruit preservative. controlled delivery of fertilizers, pesticides, and insecticides. Increase in the auxin concentration and urea release in the soil, germination capacity, root length and activity, and seedling height	[46,47,48]
Industrial application	Functional materials: Graphitic carbon nanocapsules/composites, tungsten carbide chitin whiskers, etc. are used in the production of micro-electrochemical systems and 3D networks	[49,50,51]
	Electrolyte: Sulfuric acid and chitosan combination has the ability to discharge high voltage Chitosan provides ionic conductivity and can be used in the production of solid-state batteries Photography: fixing agent for color prints	[52,53,54,55,56,57,58,59]
	Paper manufacture: Production of filter papers, water-resistant papers, biodegrading packages, water-resistant papers	[59,60,61,62,63]
	Enzyme carrier: immobilizing enzymes on solid materials	[64,65,66]
Construction industry	wood adhesive, fungicide, wood quality enhancer, and preservative	[67,68,69]
Waste treatment	Flocculating, and negative charge (chelating agent), for dye, heavy metal ions removal and decontamination. Used for various processing plants such as whey, dairy, poultry, and seafood processing plants	[70,71,72,73,74,75]

**Table 2:** Summary of different chitin extraction techniques.

Extraction Techniques	Process Conditions	Advantages	Disadvantages	References
Chemical methods	Deproteinization conditions: NaOH, KOH, Na_2_SO_3_, Na_2_CO_3_ Temp: 25–100 °C, 30 min–72 h Demineralization: HCL, HNO_3_, CH_3_COOH, HCOOH Temp: 25–100 °C, 30 min–48 h Decolorization: organic solvents such as acetone, ethyl alcohol, diethyl ether Bleaching: KMnO_4_, NaCIO/H_2_O_2_; Temp: 20–60 °C, 25 min–12 h Recovery: precipitation with 5–10%NaOH Deacetylation: NaOH/KOH 30–50% *w*/*v*, Temp: 80–150 °C, Time 1–8 h	Short processing time Produces chitin with high DA% Accompanied by deacetylation Process used at industrial scale	Multistep process Deacetylation unavoidable Environmentally unfriendly generate large quantities of waste that cannot be used as human and animal nutrients. Calcium carbonate lost to waste stream	[88,104,105]
Biological and enzyme based methods	Demineralization: fermentation using lactic acid producing bacteria or lactic acid Deproteinization using enzymes (cellulases, pectinases, chitinases, lipases, papain, hemicellulases, pepsin and lysozyme produces chitooligosaccharides, lysozyme Protease deproteinization and demineralization: in (10% HCl solution at 20 °C for 30 min) at 55 °C and pH of 8.5 Combined deproteinization and demineralization: microorganisms producing proteases or proteases Protease demineralization at 25 °C for 20 min in the presence of lactic acid ratio of 1:1.1 *w*/*w* and acetic acid ratio of 1:1.2 *w*/*w*) Deproteinized with chitinase at 45 °C and a pH of 6.0 with shaking at 150 rpm Alcalase, esperase and neutrase in deproteinization, followed by deacetylation by alkaline treatment, reached the highest degrees of deacetylation with 61.0–63.7% NaOH for 14.9–16.4 h Combination of species, including Serratia marcescens and L. plantarum, increased deproteinization and demineralization activity Decoloration: acetone or organic solvent, Deacetylation: chitin deacetylase producing by bacteria Lactic acid ratio of 1:1.1 *w*/*w* and shells: acetic acid ratio of 1:1.2 *w*/*w*) had a maximum demineralization	High quality of final product Sustainable process Environmentally safe; specific, fast in action, reduces the use of energy, chemicals and/or water compared to conventional processes Regular deacetylation and MW	Long processing time (days) Process still under development enzymatic method had a higher degree of acetylation (19.4%) and viscosity than that prepared by chemical method (17.2%).	[106,107,108,109,110,111,112,113,114,115,116,117,118,119,120]
Ionic liquids	Complete dissolution followed by the selective precipitation of chitin. Treatment with [C2C1im] [CH3COO] [121]. causes swelling swell Ionic liquids 1-ethyl-3-methylimidazolium acetate [C2mim] [OAc], 1-butyl-3-methylimidazolium chloride [C4mim]Cl, [C2mim]Cl, [C2mim] [OAc], and 1-allyl-3-methylimidazolium acetate [Amim] [OAc], are effective against chitin from shrimp shells, crab shell waste, and squid pens. Combination of steam explosion and ionic liquid pretreatments for efficient utilization of fungal chitin	Scaling-up the process were successful leading to the establishment of a company 525 Solutions at industrial scale [122]. Dissolution and coagulation of the polymer combined with enzymatic hydrolysis, reduces its crystallinity, making the polymer more accessible to the enzyme	Harsh totally dissolves chitin Toxicity and nonbiodegradability DESs are the ability to perform a three-step process in single step, including demineralization, deproteinization and chitin dissolution	[121,122,123,124,125,126,127,128]
Deep eutectic solvents	Demineralization, deproteinization and chitin dissolution perform a three-step process in single step Mixture of hydrogen bond acceptor (HBA) and a hydrogen bond donor (HBD), choline chloride (ChCl) is commonly used as an HBA, while HBDs include lactic acid, malonic acid, and citric acid 150 °C Incubating different ratio mixtures of DESs (ChCl/citric acid, ChCl/L-lactic acid, and ChCl/malic acid) with chitin sources at temperatures between 50–150 °C for 2–6 h DES plus Microwave: DES ratios of 1:5, 1:10, and 1:20. Next, the mixture was heated under 700 W microwave irradiation (Haier MZC-2070M1) for different durations of time (1, 3, 7, and 9 min) Demineralization was carried out by the malic acids. When choline chloride–malic acid was applied to the shrimp shells, minerals, which are mostly in the form of crystalline CaCO_3_, were removed by the malic acid, leaving the proteins and chitin. The spacing between the chitin–protein fibers was filled with proteins and minerals; thus, the removal of minerals resulted in a weakening of the linkages within the inner structural organization of the shrimp shells. Since the minerals are removed by the malic acids, in order to conduct demineralization, one component of the DESs used in the chitin extraction should be an acid.	Single step for simultaneous removal of protein and minerals Demineralization, deproteinization and chitin dissolution perform a three-step process in single step Low melting temperature, non-flammability, highly chemical and thermal stability and superior biodegradability. No deacetylation Solvent recycling possible	High solvent viscosity causes difficulty at large scale DESs are a new class of ionic liquid analogues derived from inexpensive commercially available raw materials with a melting point lower than that of each individual component. DESs are biodegradable, cheap and easy to produce	[129,130,131,132,133,134]
Ultrasound extraction	Ultrasound’s cavitation effect solubilizes protein associated with chitin, dissociates covalent bonds in polymer chains and disperses aggregates Uses high-intensity Ultrasound signals at 750 W power and 20 kHz ± 50 Hz operating frequency to enhance the efficiency of extraction of chitin,	Reduces the extraction time and avoids the requirement of high temperatures.		[135,136,137]
Microwave-assisted extraction	Microwave heating involves two main mechanisms: (i) dipolar polarization and (ii) ionic conduction Increasing the microwave irradiation to 130 watts of power for 15 min resulted in high deproteinization (11.46%) and a low ash content (5.4%) at 700 °C for 2 h using 50% of NaOH solution in a power range of 500–650 W resulted in a low DDA, and the deacetylation reaction was more than 80% completed after 10 min. MAE allowed the production of chitosan with medium and high MW (300–360 kDa).	Fast deacetylation of chitosan in 24 min, compared to conventional heating method that requires 6–7 h Upscaling possibility		[138,139,140,141,142]
